# Bioremediation of Cadmium Toxicity in Wheat (*Triticum aestivum* L.) Plants Primed with L-Proline, *Bacillus subtilis* and *Aspergillus niger*

**DOI:** 10.3390/ijerph191912683

**Published:** 2022-10-04

**Authors:** Sarmad Bashir, Sadia Javed, Khalid Mashay Al-Anazi, Mohammad Abul Farah, Sajad Ali

**Affiliations:** 1 Department of Biochemistry, Government College University, Faisalabad 38000, Pakistan; 2 Department of Zoology, College of Science, King Saud University, Riyadh 11451, Saudi Arabia; 3 Department of Biotechnology, Yeungnam University, Gyeongsan 38541, Korea

**Keywords:** bio-stimulation, heavy metals, abiotic stress, plants, soils, microorganisms, cereal crops

## Abstract

Cadmium toxicity is one of the deleterious abiotic factors that reduce wheat production. Two different cultivars (Akbar and Dilkash) were compared for their cadmium (0, 40 and 80 mg/kg) tolerance and responses towards *Bacillus subtilis* NA2, *Aspergillus niger* PMI-118 and L-proline. Both microbes were tested for heavy metal tolerance and production of various plant hormones and biological active enzyme characteristics under normal and cadmium stress. A completely randomized design (two cultivars × four treatments × three cadmium levels × three replicates) was adopted using distilled water as a control. The growth promotion potential of these strains under cadmium stress was determined by N-fixation, IAA synthesis, P-solubilization, amylase and proteases production. A pot experiment under controlled conditions was conducted to evaluate the effect of bacteria, fungi, and L-proline under cadmium stress. It was indicated from the result that plant biomass (46.43%), shoot length (22.40%), root length (25.06%), chlorophyll (17.17%), total sugars (27.07%), total proteins (86.01%) and ascorbic acid (83.27%) were improved with inoculation under control and cadmium stress. The accumulation of total flavonoids (48.64%), total phenolics (24.88%), hydrogen peroxide (53.96%) and activities of antioxidant enzymes CAT (26.37%) and APX (43.71%) were reduced in the plants treated with bacteria, fungi and L-proline than those under control. With parallel aids, *Bacillus subtilis* NA2 showed a higher cadmium tolerance and plant growth stability as compared to *Aspergillus niger* PMI-118 and L-proline and may be adopted in the future.

## 1. Introduction

Wheat is an important staple food in Pakistan and occupies the largest cultivated land area for a single crop [1]. It is sown on an area of approximately 22.04 million hectares and approximately 25.21 million tons of wheat is harvested [2] each year. The country is ranked 6th globally for wheat production and 8th for the total cultivated area. Punjab ranks high in wheat production and is often referred to as the capital province of Pakistan’s wheat production. The province contributes approximately 77% of the wheat produced in the country. Ironically, Pakistan imported about 1453 tones (239,221 USD) between 1991 and 2007. 

Then, after a period of three years, Pakistan’s wheat export improved over imports and this was attributed to limited development, whereas now in Pakistan, the export ratios measure greater than imports [3]. 

This suggests that wheat production does not translate to a high yield due to some growth limiting factors. The major constraint to wheat production in Pakistan is salinity, heavy metals and drought which are common in arid and semi-arid areas. These factors have been widely reported to have negative effects on plant growth and yield [4]. The deleterious effect of drought can be reduced through proper irrigation schedules and management. Heavy metals, especially cadmium, however, pose the greatest stress for crop production [5,6]. The plant devises various mechanisms to overcome heavy metal stress. Plants deal with stress from metals through either the modulation of phytohormones, or through biochemical, physiological and antioxidant mechanisms [7,8]. Moreover, metal stress could be controlled by modifying management practices, planting more metal tolerant crops, leaching the soil, a use of good quality irrigation water and the use of biological methods. However, despite all of these efforts, cadmium stress has remarkably reduced plant growth and yield, thereby leading to reduced returns on investment. 

Microorganisms, including fungi and bacteria, have been reported to remediate heavy metals from water and soil through bioaccumulation and biosorption. Many attempts were, therefore, made to apply bacteria and fungi to plants with heavy metals to obtain a higher tolerance and metal stress amelioration [9]. The utilization of biomass from fungi, algae and bacteria as an absorbent medium to remove heavy metals has garnered academic interest in the last ten years [10]. The capacity of renewable biomass from different microorganisms to act as a bio trap for heavy metals may prove to be an environmentally beneficial alternative to physicochemical remediation procedures [11]. Potentially less expensive alternatives to the traditional adsorbents could be provided by the use of microbial adsorbents, such as bacteria, fungi, algae and various agricultural wastes, which have emerged as eco-friendly and efficient material options [11,12]. 

Plant growth promoting rhizobacteria (PGPR) and fungi (PGPF) exhibit plant growth by promoting characteristics that enhance plant tolerance to environmental stresses. Several studies have reported the beneficial role of PGPR, PGPF and various chemicals in improving plant growth under cadmium conditions [6,13,14,15]. The various plant growth promoting characteristics of the PGPR line production of phytohormones, such as indole-3-acetic acid (IAA), 1-aminocyclopropane-1-carboxylate (ACC) deaminase, exopolysaccharides, volatile organic compounds, fixing atmospheric nitrogen and solubilization of mineral phosphate and potassium could contribute to alleviating cadmium stress [6,13,16]. 

The important roles of these advantageous microbes are to provide nutrients to crops, prompting plant growth specifically to produce phytohormones, for the biocontrol of phytopathogens, to improve soil structure, for the bioaccumulation of inorganic compounds and for the bioremediation of metal contaminated soils. The interface between the advantageous soil microbes and plants decide the plant health and soil fertility [17,18]. The idea of sustainable agriculture has been a consequence of utilizing the rhizosphere bacteria and fungi to facilitate the plant’s nutrient uptake and the solubilization of fixed nutrients such as phosphorus. It is the requirement of time needed to decrease the agricultural inputs through the interaction of beneficial microorganisms for an improved and sustainable agriculture [19]. The utilization of plant growth promoting rhizobacteria (PGPR) and fungi (PGPF) is a well-known approach to enhance the crop yield in metal contaminated soils. Rhizobia are extensively used to increase the growth of leguminous crops due to their capability to secure atmospheric nitrogen. Moreover, the symbiosis between plants and rhizobia is also affected by heavy metals. The consequence of the single inoculation with rhizobia can be enhanced by co-inoculating bacteria with various capacities [19].

PGPRs and PGPFs produce antioxidant defense enzymes. These enzymes play a major role in the plant adaptation to cadmium stress by scavenging the reactive oxygen species (ROS). PGPRs and PGPFs also help to convert inorganic phosphate from an insoluble form to a plant available form. PGPRs involved in the production of auxins (acetic acid) help to promote the seed germination [20]. In indirect bio-control mechanisms, a plant’s defense mechanism is induced by various incentive reagents. It is the capacity of plants to enhance their defense against a wide range abiotic stress conditions with the appropriate reagents. An induced systemic tolerance is induced by using various PGPRs [21].

Peroxidase is also an important plant defense enzyme that is involved in various plant defense mechanisms, such as oxygen reactive species quenching, oxidative cross linking and lignin synthesis. Polyphenol oxidases (PPOs) use molecular oxygen and cause the oxidation of ortho biphenolic and mono phenolic compounds. It was found that by seed priming with PGPRs and PGPFs, this induced various defense mechanisms and through this they found the rising level of peroxidase and polyphenol peroxidase in wheat plants. These are also involved in increasing the levels of IAA. Catalases are also produced in response to pathogens as they are involved in scavenging activities of the oxygen reactive species that are produced by plants in response to pathogens, such as hydroxyl radicles and hydrogen peroxide. This enzyme is involved in the decomposition of hydrogen peroxide and inhibits tissue narcotization [20,21].

Therefore, utilizing the potential of PGPRs, PGPFs and bioactive chemicals to alleviate cadmium is a promising approach that could greatly improve wheat growth under metal stress conditions. Moreover, it has been reported that PGPRs, PGPFs, proline, ascorbic acid and fertilizer improved plant growth [19]. This study was conducted in order to determine the effect of PGPRs, PGPFs, and L-proline on the wheat growth physiology, the biochemical parameters and the antioxidant enzyme activities under cadmium contaminated soil. This may lead to the formulation of eco-friendly biofertilizers that will be used for wheat production in Pakistan.

## 2. Materials and Methods

### 2.1. Microbial Culture Acquisition and Plant Growth Promoting Strains

Microbial cultures of *Bacillus subtilis* NA2 and *Aspergillus niger* PMI-118 were acquisitioned from Plant Microbe Interaction Lab, Department of Botany, Government College University Faisalabad, 38000, Pakistan. These two microbes were selected based on their bioremediation and plant growth promoting potential under heavy metal, especially cadmium, conditions. The IAA was measured by following the Salkowski reagent method [22] by growing the bacterial isolates for three days in an LB medium containing 1 g/L tryptophan under normal and metal stress conditions. A standard curve was created using indole acetic acid (IAA). The concentration of auxin was measured in micrograms per milliliter of culture. The phosphate solubilization activity of the microbes was observed by growing in NBRIP [23] and Pikoviskya’s agar [24] media. The supernatants were used to determine the solubilized phosphate using the ammonium paramolybdate method. A spectrophotometer was used to measure the absorbance at 882 nm. A nitrogen free malate medium was used to check the efficiency of microbes to fix the atmospheric nitrogen [25]. Starch agar plates with Lugol’s solution (KI and I2) were used for the appearance of a clear zone around the growth, indicating a positive result for the amylase reduction. A protease enzyme assay was carried out using casein agar media. A pure bacterial isolate culture was spot inoculated on the sterile casein agar plates. The plates were incubated for three days at 28 °C ± 2. Following the incubation, a clear zone formation was observed around the microbial colonies which was an indication of the proteases production.

### 2.2. In-Vitro Cadmium Tolerance Testing of Microbes

The test was carried out in order to observe the survival of bacterial isolates under heavy metal conditions. The nutrient agar and potato dextrose agar plates were amended with salt (CdCl_2_) at different concentrations ranging from 0 mg/L to 1200 mg/L for the bacteria and fungi, respectively. The bacterial and fungal strains were streaked on the amended agar plates to check for their ability to survive under heavy metal conditions.

### 2.3. Preparation of the Microbial Cell Suspension for Seed Bio-Priming

A single colony of a bacterial and fungal strain was mixed into the nutrient broth and fungal broth media and incubated for 24–48 h and 36–72 h at 37 °C and 32 °C, respectively, to make an inoculum suspension for the seed treatment. By vortex mixing and spinning at 6000 rpm for five minutes using a sterile 15 mL centrifuge falcon tube, the bacterial cells were washed three times with sterile distilled water. Following vertexing, the absorbance (600 nm) of the cell suspension was measured with a spectrophotometer (Hitachi UH 5300, double beam spectrophotometer, Tokyo, Japan) before being diluted to 10^8^ CFU/mL with sterile PB. To ensure the colonization during the seed germination, the surface-sterilized seeds were primed in the bacterial, fungal and L-proline suspension with continuous shaking at 120 rpm for 6 h.

### 2.4. Experiment Design

Three repetitions (Treatments (4) × cadmium stress levels (3) × cultivars (2) × replicates (3) = 72) were used in a completely randomized experiment with a total of 72 pots that were designed. Four treatments were used for pot experiments and included (T0) control (sterile distilled water); (T1) *Bacillus subtilis*; (T2) *Aspergillus niger*; (T3) L. proline (Table 1). The seeds of two wheat (*Triticum aestivum* L.) cultivars (V1: Akbar; V2: Dilkash) were collected from the Ayub Agriculture Research Institute, Faisalabad, Pakistan. The soil was taken from the Botanical Garden of the Department of Botany at GC University in Faisalabad, Pakistan, and autoclaved before use. In a prior study, we described the physiochemical parameters of the soil utilized in the experiment [18]. For each pot, twelve wheat seeds were sowed in 350 g of soil. On a bench in a greenhouse with temperatures ranging from 24 °C (night) to 31 °C (day), the pots were positioned in a completely random design. The pots were watered once a day for 30 days during the experiment. The seedlings were reduced to five per pot after germination and kept in a light-controlled environment. 0, 40, and 80 mg of CdCl_2_ per kg of soil were used to maintain the cadmium stress.

### 2.5. Plant Physiological Parameters 

The plants were collected the plant root and shoot fresh weights were measured after 30 days of growth. Following the harvesting, the plant roots were rinsed in distilled water. A ruler was used to manually measure the root and shoot lengths. The dry weight of the roots and shoots was measured by drying in an oven at 60 °C for 72 h.

### 2.6. Photosynthetic Pigments

From each replicated treatment, the fresh leaf sample (0.5 g) was homogenized in 10 mL methanol (80 percent). The samples were centrifuged for 10 min at 12,000 rpm and stored at 4 °C overnight [26]. For the Chlorophyll a, b and the total carotenoid concentrations, the absorbance of the extract was determined using a UV visible spectrophotometer at 663, 645 and 480 [18].

### 2.7. Plant Biochemical Attributes

The total flavonoid contents were measured using the Zhishen method [27]. The fresh leaf sample (0.5 g) from each replicated treatment was homogenized in 10 mL methanol (80%). The samples were centrifuged for 10 min at 12,000 rpm and stored at 4 °C overnight. 1 mL of material was combined with 0.3 mL NaNO_2_ (1%) and 0.3 mL AlCl_3_ (1%). Following a period of ten minutes, 2 mL NaOH (4%) was added. A UV spectrophotometer was used to observe the absorbance of the reaction mixture at 510 nm. T estimation of the total flavonoid content was made by comparing with quercetin standard curve. Anthrone’s reagent method [28] was used to measure the total soluble sugars. One ml Anthrone’s reagent was added to 0.1 ml sample. The mixture cooled down to room temperature after 15 min of boiling. At 625 nm, the absorbance of all treated samples were measured. The Bradford method [29] was used to calculate the total soluble protein content. The absorbance was measured at 595 nm after mixing 50 µL of the sample with one ml of the Bradford reagent. By comparing the BSA standard curve with the protein content, the amount of protein in the sample was calculated. A fresh leaf sample (0.5 g) was homogenized in 10 mL of TCA (6%) to determine the ascorbic acid concentration. The extract was then combined with 2 mL of dinitrophenyl hydrazine, followed by 1 drop of thiourea. The mixture cooled down to room temperature after 15 min of boiling. Five milliliters of 80 percent H_2_SO_4_ were added to the mixture. All treated samples were measured at 530 nm and compared to a standard curve generated using ascorbic acid concentrations ranging from 10 to 100 mg/L, as described in previous studies [30]. The total hydrogen peroxide (H_2_O_2_) levels were determined using Velikova’s technique [31]. In a mixture of 0.5 mL phosphate buffer and one mL of 1 M potassium iodide, one mL supernatant was combined with 0.5 mL phosphate buffer and 1 mL of 1 M potassium iodide after filtering. The sample mixtures were vortexed well, and their absorbance was measured at 390 nm with a spectrophotometer. A standard curve was established employing tannic acid as the reference to calculate hydrogen peroxide.

### 2.8. Activity of Antioxidant Enzymes 

A sample from a fresh leaf (0.5 g) was homogenized in 10 mL potassium phosphate buffer for the enzyme extraction to determine the antioxidant enzyme activity (pH 7.8). The extract supernatant was frozen at −20 °C in an ultra-low freezer after centrifugation for 15 min at 15,000 rpm. The activity of the CAT enzyme was determined using the method given by Chance and Maehly [32]. In a 50 mL flask, we mixed 0.1 mL of the plant extract with 1 mL of 5.9 mM H_2_O_2_ and 1.9 mL of 50 mM phosphate buffer (7.0 pH). The absorbance was measured at 240 nm for two minutes at 20 s intervals. A change of 0.01 A240 Units/min was equal to one unit of CAT activity. Following this, the CAT activities were computed and represented in mg/mg of the total soluble protein (TSP). A method provided by [32] and modified by [18] was used to determine the activity of the APX enzyme. To evaluate the activity of the APX enzyme, a reaction mixture was produced (700 µL phosphate buffers (7.0 pH) + 100 µL H_2_O_2_ (5.9 mM) + 100 µL ascorbate (0.5 mM) + 100 µL enzyme extract). A spectrophotometer was used to measure the absorbance at 290 nm for 2 min at 20 s intervals. Following this, the APX activities were computed and represented in mg/mg of the total soluble protein (TSP).

### 2.9. Statistical Analysis

To assess the influence of *Bacillus subtilis* NA2, *Aspergillus niger* PMI-118 and L proline on wheat (*Triticum aestivum* L.) under cadmium stress, a three-way completely randomized analysis of variance (ANOVA) with replication was performed using CoStat V6.4 by CoHort software. The IBM SPSS Statistics software windows version 25 was used to compute the principle component analysis (PCA) and the Pearson coefficient connection among the analyzed attributes (IBM Corp, Armonk, NY, USA).

## 3. Results

### 3.1. In-Vitro Characterization of the Microbes for Metal Tolerance, Enzyme Production and PGP Traits

The bacterial (*Bacillus subtilis* NA2) and fungal (*Aspergillus niger* PMI-118) strains were analyzed using different CdCl_2_ concentrations, and found to have varying MIC values in the metal tolerance test (Table 2). The bacterium and fungus show a tolerance against the different levels of cadmium stress from 0–1200 mg/mL CdCl_2_ by differing growth patterns. The selected bacterial and fungal species were unable to produce IAA without substrates, but able to produce (*B. subtilis*; 23.46, *A. niger* 56.41 μg/mL) when subjected to the substrate tryptophan in a LB medium. A comparison was made with both the stressed (CdCl_2_ 800 mg/L) and non-stressed conditions. The IAA production was recorded as high in the non-stressed condition and the strains were also capable to produce IAA (*B. subtilis*; 11.49, *A. niger* 36.21 μg/mL) under stress conditions. Both microbes grew well on a nitrogen free medium, without a nitrogen source, by fixing the atmospheric nitrogen. When characterized as plant growth promoting for phosphate solubilization (42.2, 38.1 and 30.9 ppm), the IAA production and nitrogen fixation showed positive results for these in-vitro PGP analyses (Table 2). Both microbes were able to produce sufficient amylase and proteases. The zone diameter of the protease activity was recorded as 18 ± 1 and 12 ± 1 mm by *Bacillus subtilis* NA2 and *Aspergillus niger* PMI-118, respectively.

### 3.2. Plant Physiological Parameters Affected by the Different Treatments

**Figure 1:**
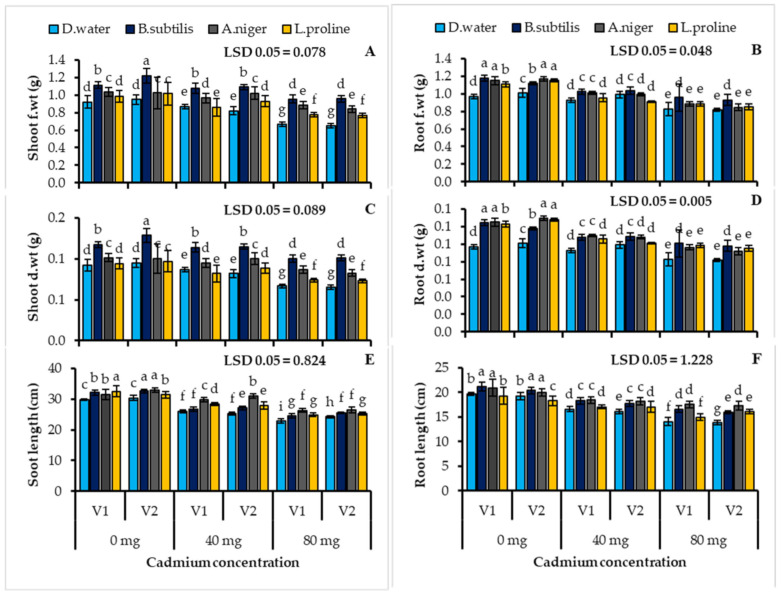
Shoot fresh weight (**A**) root fresh weight (**B**) shoot dry weight (**C**) root dry weight (**D**) shoot length (**E**) root length (**F**) of two cultivars of wheat (*Triticum aestivum* L.) treated with *Bacillus subtilis*, *Aspergillus niger* and L-proline subjected to the cadmium stress condition (Mean ± S.E.). Here V1 = AKBAR and V2 = DILKASH. The lower-case letters represent the significant difference in data.

Under cadmium stress (40, 80 mg/kg soil), the plant biomass had significantly decreased reductions in plant shoot fresh weight of for V1: AKBAR (27.54%), V2: DILKASH (31.23%) and the fresh weight for V1 (14.43%), V2 (18.87%) were observed in the untreated plants (Table 3, Figure 1). The wheat plants treated with *Bacillus subtilis*, *Aspergillus niger* and L-proline recorded the increased fresh biomass as compared to the shoot fresh weight, as *Bacillus subtilis* (V1:43.86, V2: 46.43%), *Aspergillus niger* (32.50, 28.57%), and L-proline (16.50, 17.35%) and the root fresh weight *Bacillus subtilis* (V1:15.26, V2: 13.88%), *Aspergillus niger* (6.83, 3.67%), and L proline (6.83, 4.49%) when compared with the untreated control plants (Table 3, Figure 1A). Additionally, in the stressed plants treated with microbes and chemicals, the accumulation of the plant shoots dry biomass increased significantly, as *Bacillus subtilis* (V1:50.53, V2: 54.14%), *Aspergillus niger* (29.90, 26.05%), and L-proline (10.95, 11.76%), compared with the cadmium stress control that was not treated. Moreover, the inoculation with *Bacillus subtilis* (V1:21.33, V2: 19.87%), *Aspergillus niger* (16.12, 12.69%), and L-proline (18.70, 16.10%), when compared with the untreated cadmium stress control, there was a significantly higher plant root dry weight in both wheat cultivars. (Table 3, Figure 1D).

The plant growth decreased significantly under the cadmium stress (40, 80 mg/kg soil), the reductions in the plant shoot length of V1: AKBAR (23.38%), V2: DILKASH (20.29%) and the root length V1 (28.60%), V2 (28.08%) in untreated plants were noticed (Table 3, Figure 1). In both wheat (*Triticum aestivum* L.) cultivars, the shoot and root lengths were improved by the bacterial, fungal, and chemical treatments under cadmium stress. The inoculation with *Bacillus subtilis* (V1: 7.45, V2: 5.36%), *Aspergillus niger* (15.33, 9.35%), and L-proline (8.91, 4.13%), compared to the untreated stressed control, the stressed plants of both wheat cultivars had longer shoots (Table 3, Figure 1E). The treatment with *Bacillus* significantly lengthened the plant roots as *Bacillus subtilis* (V1: 17.77, V2: 15.42%), *Aspergillus niger* (24.64, 25.06%) and L-proline (6.40, 16.14%) compared to the untreated stressed plants in both cultivars (Table 3, Figure 1F).

### 3.3. Plant Photosynthetic Pigment Contents

**Figure 2:**
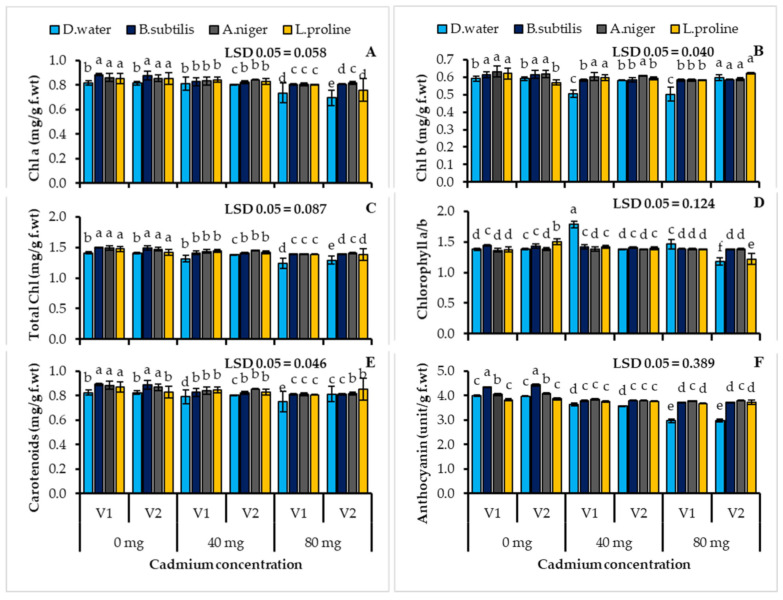
Chlorophyll a (**A**) chlorophyll b (**B**) total chlorophyll (**C**) chlorophyll a/b (**D**) carotenoids (**E**) and anthocyanin (**F**) contents of two cultivars of wheat (*Triticum aestivum* L.) treated with *Bacillus subtilis*, *Aspergillus niger* and L-proline subjected to the cadmium stress conditions (Mean ± S.E.). Here V1 = AKBAR and V2 = DILKASH. The lower-case letters represent the significant difference in data.

Uninoculated cadmium stressed plants produced fewer photosynthetic pigments, which was followed by a significant decrease in the chlorophyll a contents of both wheat cultivars’ (V1; 9.92, V2; 14.80%), chlorophyll b (15.09, 10.04%), carotenoid (9.06, 10.25%) and anthocyanin (25.61, 24.68%) when compared with the untreated control (Table 3, Figure 2). Under both conditions, different treatments significantly (*p* ≤ 0.001) improved the chlorophyll (a, b, and total) and carotenoid contents. Under the cadmium containment soil stress, both wheat cultivars had higher chlorophyll a, b, total chlorophyll and carotenoid contents than the uninoculated plants. The determined increase in chlorophyll a with *Aspergillus niger* in V2 was (17.17%), chlorophyll b with *Aspergillus niger* in V2 was (19.03%), when compared with the uninoculated control (Table 3, Figure 2A–C). As opposed to the uninoculated control, the plants inoculated with *Bacillus subtilis* (8.11, 7.82%) showed improved carotenoid contents. (Table 3, Figure 2E). All three treatments showed significant improvements in the anthocyanin contents as *Bacillus subtilis* (24.97, 24.62%), *Aspergillus niger* (27.35, 26.81%) and L-proline (23.92, 24.41%) in both wheat cultivars, under cadmium stress conditions, when compared with the stressed plants (Table 3, Figure 2F).

### 3.4. Plant Biochemical Attributes Estimation

**Figure 3:**
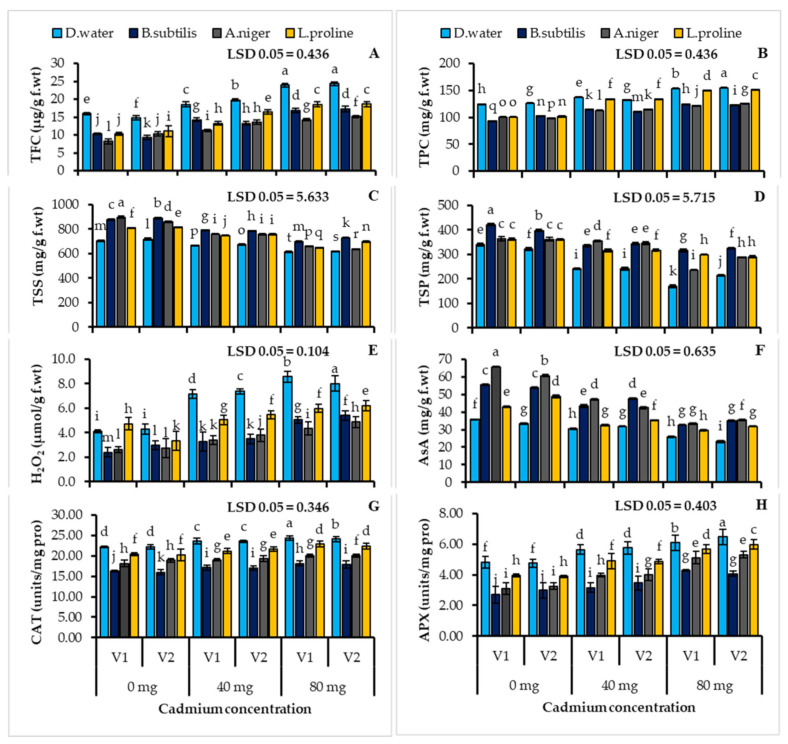
Total flavonoid contents (**A**) total phenolic contents (**B**) total soluble sugars (**C**) total soluble proteins (**D**) hydrogen peroxide (**E**) ascorbic acid (**F**) contents, catalase (**G**) ascorbate peroxidase (**H**) activity of two cultivars of wheat (*Triticum aestivum* L.) treated with *Bacillus subtilis*, *Aspergillus niger* and L-proline subjected to cadmium stress conditions (Mean ± S.E.). Here V1 = AKBAR and V2 = DILKASH. The lower-case letters represent the significant difference in data.

Plant biochemical parameters decreased significantly under the cadmium stress conditions; the reductions in the plant’s total soluble sugars (V1; 12.98, V2; 13.81%), total soluble protein (49.91, 33.65%) and ascorbic acid contents (28.07, 30.28%) were noticed in the untreated control plants (Table 3, Figure 3). The wheat plants treated with *Bacillus subtilis* (24.52, 23.82%), *Aspergillus niger* (27.07, 19.72%) and L-proline (14.74, 13.38%) have a higher level of total soluble sugars in the cadmium stressed plants of both wheat cultivars, contrasted with the untreated control (Table 3, Figure 3C). Under both control and metal stress conditions, the inoculation led to an increase in the total soluble protein contents as *Bacillus subtilis* (86.00, 52.17%), *Aspergillus niger* (38.91, 34.68%) and L-proline (75.64, 35.52%), when compared with the stress control without treatment (Table 3, Figure 3D). The plant ascorbic acid level improved significantly with *Bacillus subtilis* (26.61, 49.57%), *Aspergillus niger* (29.59, 51.29%) and L-proline (14.47, 36.71%) in both cultivars (V1, V2) with respect to the untreated control (Table 3, Figure 3F). 

The accumulation of flavonoids (V1; 50.10, V2; 64.41%), phenolics (23.27, 22.18%) and hydrogen peroxide (108.55, 86.77%) contents was observed in both wheat cultivars under the cadmium stress condition (Table 3, Figure 3). The flavonoid contents were found to have significantly decreased in both wheat cultivars when treated with *Bacillus subtilis* (29.64, 28.90%), *Aspergillus niger* (40.47, 37.67%) and L-proline (22.67, 23.42%) under the cadmium stress condition contrasted with the untreated control (Table 3, Figure 3A). The plant’s total phenolic contents was also reduced when treated with *Bacillus subtilis* (18.96, 20.52%), *Aspergillus niger* (20.67, 18.36%) and L-proline (2.22, 2.10%) when compared with the control group (Table 3, Figure 3B). The plants treated with *Bacillus subtilis* 41.20, 32.44%), *Aspergillus niger* (49.34, 39.24%) and L-proline (30.09, 22.59%) showed a significant reduction in hydrogen peroxide levels under the cadmium stress conditions when compared with the untreated stressed plants (Table 3, Figure 3E).

The antioxidant enzyme response to the cadmium stress was stimulated in the untreated plants, the enhanced catalase (10.07, 8.75%) and the ascorbate peroxidase (26.47, 35.91%) activities (Table 3, Figure 3). Both wheat cultivars showed a significantly different trend in the increase in the enzyme activities. Additionally, it was demonstrated that the inoculation of L-proline, *Aspergillus niger* and *Bacillus subtilis* reduced the plant’s enzymatic antioxidant responses to metal stress. The wheat plants treated with microbes and chemicals showed a decrease in the CAT activity as *Bacillus subtilis* (26.37, 27.87%), *Aspergillus niger* (18.21, 14.83%) and L-proline (7.95, 8.70%) (Table 3, Figure 3G) contrasted with the untreated control. Additionally, the treated plants showed a significant decrease in the APX activity as *Bacillus subtilis* (43.71, 37.37%), *Aspergillus niger* (35.40, 31.49%) and L-proline (17.54, 18.10%), in contrast to the control group (Table 3, Figure 3H).

**Figure 4:**
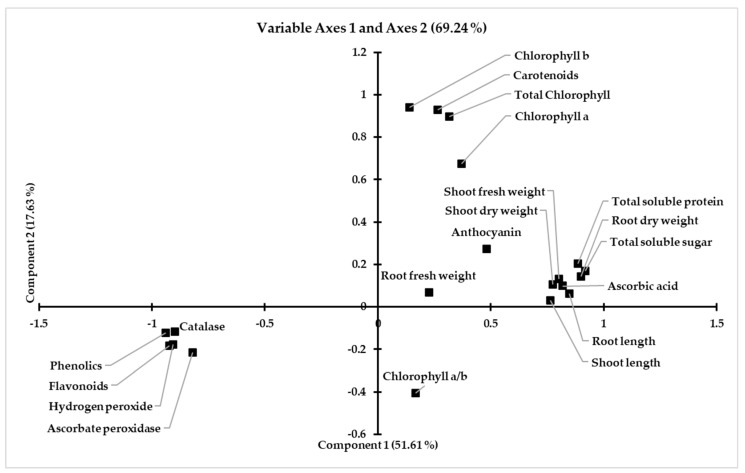
Component plot in the rotated space by the factor analysis of the studied parameters of wheat treated with bacteria, fungi and L-proline under control (0 mg/kg soil) and cadmium containment soil (40, 80 mg/kg soil) conditions.

Pearson’s correlation of all of the studied parameters is reported in (Table 4). It is evident that there is a significant positive correlation between the plant biomass and the physiological characteristics that have been studied, such as the plant length, chlorophyll, carotenoids, anthocyanin, total soluble sugars, total soluble proteins and ascorbic acid concentrations. However, the contents of flavonoids, phenolics, hydrogen peroxide and the activities of the antioxidant enzymes were negatively correlated with the shoot biomass. The studied attributes are divided into two main groups, according to the correlation studies shown in (Table 4, Figure 4) that were produced using a component analysis, and 51.61 percent of the variance was explained by the first PCA component, and 17.63 percent by the second. 

## 4. Discussion

This study investigated the effects of the addition of cadmium tolerant bacteria, fungi and L-proline on the growth, biochemical parameters and activities of antioxidant enzymes on wheat growing at different cadmium levels. The increases in soil metal are known to decrease plant growth and nutrient uptake. The in vitro laboratory screening and the plant inoculation application revealed that wheat with PGPR (*Bacillus subtilis*) PGPF (*Aspergillus niger)* and chemicals (L-proline), had higher growth parameters and physiological variables as plant biomass shoot and root length, photosynthetic content, biochemical attributes and antioxidant activities. The wheat inoculation with microbes and L-proline reduced the secondary metabolites (flavonoids and phenolics), H_2_O_2_. and the drastic effects of metal stress (Table 3, Figure 2 and Figure 3). The enhanced nutrient uptake, plant growth and development by the halotolerant bacteria, fungi and L-proline have also been previously reported in wheat, rice, meadows, cucumber, tomato and maize plants [8,33,34,35,36,37]. It is also well known that salt stress can induce nutrient imbalances in plants that lead to stunted growth [38,39].

Furthermore, the *Bacillus subtilis*, *Aspergillus niger* and L-Proline induced a cadmium metal tolerance and improved the growth in both the Akbar and Dilkash wheat cultivars. The wheat growth under stress conditions was improved by the inoculation of the ACC deaminase-producing bacteria *Bacillus cereus* strain Y5, Bacillus sp. Y14 and *Bacillus subtilis* strain Y16 [40]. Under conditions of weather, salt and temperature stress, various bacterial and fungal species significantly affect plant growth. The results of the current study, which involved inoculating wheat with PGPRs (*Bacillus subtilis*, *Aspergillus niger*) and L-proline, observed a significant improvement in the metal-treated plants [14,15,41]. Together, these studies show that PGPRs and L-proline have a favorable effect on plant growth and improvement under control and cadmium stress.

The secondary metabolite accumulation, flavonoids, phenolics and hydrogen peroxide accumulation in plants is an indication of good stress responses or heavy metal tolerance because it mediates the osmotic adjustment, protects the cytoplasmic macromolecules from dehydration and functions as an oxidative stress scavenger. [6,14,42]. Hydroxyl radicals and reactive oxygen species (ROS) rise in wheat under heavy metal like cadmium containment soil conditions [38,43] and cause cellular toxicity in plants growing under high stress [44,45], the antioxidant systems are immensely important in resolving such subcellular damage and protecting plants against these abiotic environmental stress conditions [39,46]. The antioxidant compounds are among the measures of a plant’s stress tolerance. Catalase and ascorbate peroxidase enzymes are low molecular weight scavengers produced by plants to provide tolerance to heavy metal stress [40]. However, the majority of plant species do not produce enough antioxidant compounds to deal with the severe effects of biotic and abiotic stress. The rise in activities of the antioxidant enzyme, catalase, ascorbate peroxidase and others indicate that heavy metal-induced oxidative stress is being mitigated in plants [35]. Gladiolus plants can withstand environmental stress by producing more proline, POX and other defensive enzymes [47,48].

Taken together, the above findings show that the PGPR strains and L-proline alleviate the consequences of cd stress on wheat crops. Inoculation within the endophytic bacterium *(Bacillus subtilis*), fungus (*Aspergillus niger*) and L-proline was significantly beneficial, though to varying degrees.

This study paves the way for further research into the genetic mechanisms underlying the PGPR-mediated initiation of cadmium tolerance in wheat and other field crops. Understanding the molecular interactions between plant and bacterial strains may benefit from research on the expression profiles of metal-responsive genetic makeup in cereals in responding to PGPR inoculation. To protect plants from soil-borne diseases, increase their tolerance to metal stress, and promote plant growth and development, the inculcation with PGPRs, PGPFs and biochemicals enhances various molecular mechanisms in plants. The synthesis of phytohormones and antimicrobial activity metabolites is increased, plant nutrients are more readily available, stress-induced ethylene production is reduced, and the systemic resistance is induced, among other mechanisms. [48]. According to earlier research, the two main mechanisms involved in the reduction of heavy metal stresses may be the regulatory oversight of mineral uptake and the rise in antioxidant enzyme activities.

There is little information currently available on the identification, screening, application and production of potential phytohormone and enzyme-producing plant growth-promoting bacteria [49,50]. Our findings show that even under cadmium conditions, the PGPR strains have shown promising PGP characteristics which could be used as biofertilizers to increase soil fertility and plant growth.

## 5. Conclusions

In both wheat genotypes (Akbar and Dilkash) under cadmium stress, the inoculation of the PGPR, PGPF and L-proline had a conclusive effect on the alteration of toxic elements, improved resistance and optimistic biochemical responses. These results make it possible to investigate plant-microbes and L-proline interactions in the presence of heavy metals, particularly cadmium. In contrast to the wheat variety Dilkash inoculated with *Bacillus subtilis* and *Aspergillus niger*, the cultivar Akbar inoculated with *Bacillus subtilis* demonstrated a higher metal tolerance as well as an improved plant growth, physiology, total protein, total sugars and ascorbic acid accumulation up to 46.43, 25.06, 86.01, 27.07, 83.27%, respectively. The catalase and ascorbate peroxidase activities were higher in untreated plant groups, when compared with those treated with microbes and L-proline. Our findings suggest that, for ameliorating cadmium stress (40 and 80 mg/kg of soil) and increasing wheat growth in heavy metal soils, the application of *Bacillus subtilis* is relatively better as compared to the *Aspergillus niger* and L-proline. Still, both microbial and chemical applications could be used as sustainable solutions. The degradation capacity of microbes makes them the most important group of the organisms in terms of public health engineering. Microbes are critical to agricultural sectors as an army to combat the diseases and for the bioremediation of agricultural wastes.

In future, this plant microbe interaction may be utilized to improve the plant growth for the production of food, fiber, biofuels and key metabolites under environmental stresses. 

## Figures and Tables

**Table 1:** Greenhouse experimental conditions for wheat (*Triticum aestivum* L.) plants inoculated with *Bacillus subtilis*, *Aspergillus niger* and L proline under control and cadmium stress.

Parameters	Details
**Treatments**	
Control (T0)	Sterilized distilled water
Bacterial (T1)	*Bacillus subtilis* NA2
Fungal (T2)	*Aspergillus niger* PMI-118
Chemical (T3)	L-proline
**Wheat cultivars**	
Wheat (V1)	Akbar
Wheat (V2)	Dilkash
**Cadmium stress levels**	
Cadmium CdCl_2_ (S0)	0 mg/kg of soil
Cadmium CdCl_2_ (S1)	40 mg/kg of soil
Cadmium CdCl_2_ (S2)	80 mg/kg of soil
**Growth conditions**	
Temperature (Day)	31 °C
Temperature (Night)	24 °C
Soil (Per Pod)	350 g

**Table 2:** Characterization of the microbes for the metal tolerance, enzyme production and PGP traits.

Characteristics	*B. subtilis*	*A. niger*
IAA Synthesis		
IAA (without tryptophan)	-	-
IAA (with tryptophan)	23.46 (μg/mL)	56.41 (μg/mL)
IAA (CdCl_2_ 800 mg/L + tryptophan)	14.49 (μg/mL)	36.21 (μg/mL)
Nitrogen Fixation		
N-free semi-solid media	+	+
N-free malate agar media	+++	+++
Ammonia Production		
Nessler’s reagent	+++	+++
Ammonia Production (μmol/mL)	6.89	5.34
Phosphate solubilization		
NBRIP media	+++	-
Pikovaskkaya’s agar media	+	++
Phosphate solubilization (ppm)	42.2	38.1
Enzyme Production		
Amylase	+++	+++
Proteases (Zone mm)	18 ± 1	12 ± 1
CdCl_2_ Tolerance		
0 mg/L	+++	+++
200 mg/L	+++	+++
400 mg/L	+++	+++
600 mg/L	+++	++
700 mg/L	++	++
800 mg/L	++	+
900 mg/L	++	-
1000 mg/L	+	-
1100 mg/L	-	-
1200 mg/L	-	-

-, +, ++, and +++ = negative, positive, high positive and strong positive, respectively.

**Table 3:** Statistically mean squares with significant level from the three-way analysis of variance for various physiological and biochemical parameters of the wheat (*Triticum aestivum* L.) plants inoculated with *Bacillus subtilis*, *Aspergillus niger* and L-proline under the control (0 mg/kg soil) and cadmium stress (40, 80 mg/kg soil) conditions.

**Source**	**df**	**SFW**	**SDW**	**RFW**	**RDW**	**Root L**
**Variety (V)**	1	0.281 ns	0.296 ns	0.029 ns	0.039 ns	0.787 ns
**Stress (S)**	2	21.69 ***	22.22 ***	47.78 ***	49.58 ***	30.08 ***
**Treatment (T)**	3	15.34 ***	26.20 ***	6.693 ***	17.33 ***	5.461 ***
**V × S**	2	0.339 ns	0.354 ns	0.439	0.454 ns	0.306 ns
**V × T**	3	0.189 ns	0.203 ns	0.359 ns	0.342 ns	0.109 ns
**S × T**	6	0.271 ns	0.286 ns	1.527 ns	1.800 ns	0.624 ns
**V × S × T**	6	0.209 ns	0.219 ns	0.360 ns	0.373 ns	0.095 ns
**Source**	**df**	**Shoot L**	**Chl a**	**Chl b**	**Total Chl**	**Chl a/b**
**Variety (V)**	1	1.368 ns	0.199 ns	0.889 ns	0.017 ns	2.067 ns
**Stress (S)**	2	132.6 ***	4.401 *	1.406 ns	3.524 ns	1.790 ns
**Treatment (T)**	3	15.72 ***	1.697 ns	1.875 ns	2.209 *	0.282 ns
**V × S**	2	0.297 ns	0.057 ns	1.194 ns	0.150 ns	1.198 ns
**V × T**	3	0.715 ns	0.065 ns	1.061 ns	0.196 ns	1.633 ns
**S × T**	6	1.736 ns	0.267 ns	0.298 ns	0.162 ns	0.887 ns
**V × S × T**	6	0.462 ns	0.050 ns	0.269 ns	0.021 ns	0.528 ns
**Source**	**df**	**Carotenoids**	**Anthocyanin**	**Flavonoids**	**Phenolics**	**TSS**
**Variety (V)**	1	0.105 ns	0.003 ns	18.07 ***	7.809 **	6.668 *
**Stress (S)**	2	3.288 *	4.961 *	760.3 ***	5317 ***	2112 ***
**Treatment (T)**	3	1.603 ns	2.033 ns	441.9 ***	2617 ***	773.7 ***
**V × S**	2	0.605 ns	0.015 ns	5.884 **	27.48 ***	6.047 **
**V × T**	3	0.178 ns	0.008 ns	12.54 ***	3.295 *	19.39 ***
**S × T**	6	0.252 ns	0.824 ns	7.946 ***	224.1 ***	69.66 ***
**V × S × T**	6	0.127 ns	0.002 ns	4.275 **	22.18 ***	6.147 ***
**Source**	**df**	**TSP**	**H_2_O_2_**	**AsA**	**CAT**	**APX**
**Variety (V)**	1	5.152 *	7.621 **	2.373 ns	0.008 ns	0.177 ns
**Stress (S)**	2	808.3 ***	1756 ***	2358 ***	128.7 ***	39.32 ***
**Treatment (T)**	3	458.4 ***	1429 ***	1231 ***	98.81 ***	12.33 ***
**V × S**	2	26.57 ***	12.40 ***	6.416 **	0.384 ns	0.441 ns
**V × T**	3	4.412 **	17.37 ***	39.60 ***	0.418 ns	0.054 ns
**S × T**	6	42.26 ***	98.01 ***	131.3 ***	6.476 ***	0.251 ns
**V × S × T**	6	8.001 ***	21.75 ***	15.84 ***	0.444 ns	0.333 ns

ns = non-significant; *, ** and *** = significant at 0.05, 0.01 and 0.001 levels, respectively.

**Table 4:** Pearson’s coefficient correlation values of the estimated attributes of wheat (*Triticum aestivum* L.) inoculated with *Bacillus subtilis*, *Aspergillus niger* and L-proline, showing significant differences under cadmium stress.

	SFW	SDW	RFW	RDW	SL	RL	Chla	Chlb	TChl	Chlab	Caro	Antho	Flavo	Pheno	TSS	TSP	H_2_O_2_	AsA	CAT	APX
**SFW**	1																			
**SDW**	0.981	1																		
**RFW**	0.324	0.279	1																	
**RDW**	0.526	0.476	0.242	1																
**SL**	0.511	0.490	0.121	0.628	1															
**RL**	0.616	0.543	0.201	0.728	0.698	1														
**Chl a**	0.413	0.383	0.092	0.324	0.290	0.329	1													
**Chl b**	0.217	0.190	0.099	0.233	0.165	0.205	0.508	1												
**TChl**	0.381	0.348	0.109	0.329	0.274	0.319	0.914	0.812	1											
**Chlab**	0.115	0.114	−0.009	0.049	0.067	0.064	0.310	−0.616	−0.079	1										
**Caro**	0.335	0.303	0.130	0.320	0.222	0.284	0.731	0.886	0.910	−0.245	1									
**Antho**	0.403	0.390	0.133	0.411	0.431	0.395	0.212	0.333	0.300	−0.098	0.389	1								
**Flavo**	−0.688	−0.631	−0.154	−0.793	−0.695	−0.812	−0.469	−0.304	−0.460	−0.085	−0.404	−0.459	1							
**Pheno**	−0.725	−0.710	−0.161	−0.787	−0.699	−0.775	−0.436	−0.251	−0.413	−0.100	−0.354	−0.426	0.903	1						
**TSS**	0.7109	0.688	0.188	0.805	0.663	0.785	0.429	0.294	0.428	0.063	0.420	0.436	−0.866	−0.861	1					
**TSP**	0.703	0.683	0.102	0.732	0.656	0.756	0.412	0.340	0.438	−0.008	0.402	0.500	−0.852	−0.817	0.866	1				
**H_2_O_2_**	−0.707	−0.686	−0.152	−0.714	−0.703	−0.742	−0.406	−0.324	−0.426	0.011	−0.371	−0.477	0.913	0.877	−0.820	−0.875	1			
**AsA**	0.653	0.628	0.224	0.788	0.668	0.744	0.402	0.273	0.400	0.062	0.385	0.396	−0.865	−0.885	0.921	0.786	−0.847	1		
**CAT**	−0.756	−0.763	−0.095	−0.682	−0.640	−0.733	−0.358	−0.265	−0.367	−0.008	−0.335	−0.458	0.831	0.900	−0.824	−0.835	0.857	−0.808	1	
**APX**	−0.632	−0.605	−0.221	−0.679	−0.590	−0.699	−0.448	−0.294	−0.441	−0.060	−0.431	−0.416	0.793	0.796	−0.796	−0.761	0.771	−0.764	0.692	1

SL = shoot length; RL = root length; SFW = shoot fresh weight; SDW = shoot dry weight; RFW = root fresh weight; RDW = root dry weight; Chl a = chlorophyll a; T Chl = total chlorophyll; Chl b = Chlorophyll b; Antho = anthocyanin; Caro = Carotenoids; Flav = flavonoids; AsA = ascorbic acid; TSS = total soluble sugars; Pheno = Phenolics; TSP = total soluble protein; H_2_O_2_ = hydrogen peroxide; CAT = catalase; APX = ascorbate peroxidase.

## Data Availability

The dataset generated during and/or analyzed during the current study are available from the corresponding author on resealable request.
